# Acute Management of Cocaine-Associated Methaemoglobinaemia

**DOI:** 10.1155/2011/136396

**Published:** 2011-12-29

**Authors:** Immo Weichert

**Affiliations:** Acute Medicine Unit, Royal Alexandra Hospital, Corsebar Road, Paisley PA2 9PN, UK

## Abstract

Methaemoglobinaemia is a potentially life-threatening complication of problem drug use. This is a case report of a 29-year-old man who presented himself cyanosed after a cocaine binge. It highlights the diagnosis and management of this condition from an acute medical perspective.

## 1. Case Presentation

A 29-year-old man presented himself to the emergency department after noticing that his lips and fingers had turned blue. He had been on a cocaine binge preceding his admission. He estimated that he had been taking at least 14 grams over a period of five days. He felt short of breath on moderate exertion but denied chest pain or any other systemic symptoms. He had been using cocaine regularly for at least ten years, always via the nasal passageways, never injected, and denied taking any other recreational drugs. He was not on any prescription or over-the-counter medications. His only past medical history was of alcohol dependence, though he had not drunk in excess for at least ten months. There was no significant family history.

He was alert and coherent, but slightly anxious. He was apyrexial, his noninvasive blood pressure was 135/96 mmHg, and his heart rate was 85 beats/min. He was tachypnoeic with a respiratory rate of 18/min. There was marked cyanosis of his fingers and lips, and he was, immediately upon arrival in the emergency department, commenced on high flow oxygen. His saturation measured by pulse oximetry (SO_2_) was 86% on 15 L of oxygen given via a nonrebreathing mask. Arterial blood gasses showed H+ 50.9 nmol/L, pCO_2_ 5.8 kPa, pO_2_ 72.3 kPa, lactate 0.7 mmol/L, bicarbonate 26.0 mmol/L, CO Hb 0.0%. His oxygen saturation on the blood gas (O_2_ Hb) was 66.6% and his methaemoglobin (Met Hb) level 32.9%. Chest X-Ray, ECG, full blood count, and routine biochemistry were unremarkable.

A diagnosis of methaemoglobinaemia was made, and he received one dose of methylene blue (1 mg/kg) intravenously over five minutes. He was transferred to the high dependency unit where he continued to receive high flow oxygen and was closely monitored.

His methaemoglobin levels, as well as his saturation, improved steadily over the next hours (Table 1). He made a full recovery and was discharged home the same evening after review by the local drug addiction liaison service with no further followup.

## 2. Discussion

Acquired methaemoglobinaemia is a well-recognized though still relatively rare complication of cocaine use. It is attributed to adulterants, cheaper substances that are being mixed with the pure cocaine base to increase the profits from selling the drug. Local anaesthetics are being used as they will produce a similar sensation when applied to mucosal surfaces as unadulterated cocaine [1]. Benzocaine is often implied in acquired methaemoglobinaemia, though its use as adulterant does vary between countries. Benzocaine is the most frequently found cutting agent in the UK [2] while a recent French study failed to show its significant use there [3].

Methaemoglobinaemia is caused by oxidation of the iron molecule in the heme group to the ferric state (Fe 3+). This renders it unable to carry oxygen and causes a functional anaemia. Blood normally can contain about 1% of methaemoglobin. Substances that put high oxidative stresses onto haemoglobin will increase the level above normal. Well-recognized causes for this are local anaesthetics (especially prilocaine, benzocaine), antibiotics (dapsone, trimethoprim, sulfonamides), aniline dyes, and metoclopramide, as well as nitrates. Blood that contains significant amounts of methaemoglobin has a characteristic dark reddish-brown colour. Above concentrations of 1.5 g/dL (Met Hb 10–15%), this will cause cyanosis even in the absence of elevated deoxyhaemoglobin levels. At relatively low levels this will lead to a cyanotic but asymptomatic patient. Typically, the cyanosis will not improve after increasing the oxygen supply. Symptoms in otherwise healthy individuals will start at methaemoglobin levels of 30% or more. These will be related to oxygen deficiency in organs with high demands, that is, the cardiovascular and central nervous system. Patients will experience headaches, lightheadedness, anxiety, dyspnoea, palpitations, and somnolence. Significant toxicity will occur at methaemoglobin levels of 50% and over. This will include cardiac arrhythmias, delirium, seizures, coma as well as a profound metabolic acidosis. Death will occur at concentrations above 60% [4], though in patients with comorbidities this can occur at much lower levels. 

Methaemoglobin interferes with traditional (not multiwavelength) pulse oximetry. In patients with relatively low methaemoglobin levels this will give falsely low oxygen saturations. Paradoxically, it will also lead to falsely elevated oxygen saturations in the presence of significant concentrations [5]. Arterial blood gasses will often show normal PaO_2_ levels but falsely low O_2_ saturations. This difference between the oxygen saturation from the blood gas analysis and the saturation, as measured by the saturation probe, is commonly referred to as the “saturation gap.” It suggests the presence of a haemoglobin derivate that is not transporting oxygen. In the presence of cyanosis, this is highly suggestive of methaemoglobinaemia [6]. In severe cases there will be a significant acidosis. Lactate levels, urea, and electrolytes, as well as a creatinine kinase and 12 lead ECG, can help to assess the degree of tissue hypoperfusion and end-organ damage. These investigations will also point towards other cocaine-related toxicities. A pregnancy test should be performed in women of childbearing age. A urine screen for drug metabolites can help in unclear cases. Sometimes a blood film will show Heinz bodies in the erythrocytes, inclusions of denatured haemoglobin [7]. Good history taking is paramount to identify the possible offending agent and to distinguish primary from secondary causes of methaemoglobinaemia. In case of internal concealment of cocaine (“body packers”), oral purgation and sometimes surgical removal may be necessary [8]. Cases with severe toxicity, decreased level of consciousness, or multiple organ failure should be treated after obtaining advice from the National Poisons Information Service or similar and in a high dependency or intensive care environment.

Healthy and asymptomatic patients with low levels of methaemoglobin may only need administration of high flow oxygen and observation. As long as the offending agent has been removed, Met Hb levels will return to normal within 36 hours [9]. Methylene blue (methylthioninium chloride) is the antidote of choice if methaemoglobin levels have reached 30% or if there is symptomatic hypoxaemia or ischaemia [10]. It leads to the reduction of methaemoglobin both via the NADPH-methaemoglobin reductase as well as through its own intermediary, leucomethylene blue [11]. The initial dose is 1 mg/kg bodyweight given intravenously over five minutes. Effects will be measurable within 30 minutes to one hour. In severe toxicity, this may have to be repeated. Failure to respond to methylene blue suggests G6PD deficiency where it can also cause profound haemolysis. Repeated or high doses can lead to a paradoxical increase in methaemoglobin [12]. Its effects during pregnancy are unsure, and potential risks of teratoxicity need to be considered [13]. It can cause blue discolouration of the urine as well as of the sclera [14]. Other rare but serious side effects are related to its MAO-inhibitor action [15], and it has been implied in the serotonin syndrome [16]. The latter is a potentially life-threatening condition caused by excessive serotonergic activity in the nervous system. Features include mental status changes, autonomic instability, and neuromuscular hyperactivity [17]. Patients who have been on selective serotonin reuptake inhibitor antidepressants (SSRIs) or clomipramine and are treated with methylene blue should be observed for CNS effects for up to four hours after administration [18]. Alternative treatments which have been successfully used in significant methaemoglobinaemia, or in patients that are unable to take methylene blue, are hyperbaric oxygen and exchange transfusions [19].

The prognosis of methaemoglobinaemia is determined by the degree of end-organ damage. Mild-to-moderate severe cases will make a complete and swift recovery.

Recurrent presentations, partial or nonresponse to methylene blue, should raise suspicion of a primary methaemoglobinaemia or G6PD deficiency and warrant further specialist assessment.

## Figures and Tables

**Table 1:** 

Time	23:16 (admission)	00:29	02:23	15:35
Met Hb in %	32.9	16.9	1.9	0.2
O_2_ Hb in %	66.6	82.4	93.1	98
SO_2_ in %	86	89	91	97
